# Wearable Contact Lens Sensor for Non-Invasive Continuous Monitoring of Intraocular Pressure

**DOI:** 10.3390/mi12020108

**Published:** 2021-01-22

**Authors:** Zhiqiang Dou, Jun Tang, Zhiduo Liu, Qigong Sun, Yang Wang, Yamin Li, Miao Yuan, Huijuan Wu, Yijun Wang, Weihua Pei, Hongda Chen

**Affiliations:** 1 The State Key Laboratory on Integrated Optoelectronics, Institute of Semiconductors, Chinese Academy of Sciences, Beijing 100083, China; douzhiqiang@semi.ac.cn (Z.D.); junt@semi.ac.cn (J.T.); liuzhiduo@semi.ac.cn (Z.L.); sunqigong@semi.ac.cn (Q.S.); wangyang@semi.ac.cn (Y.W.); liyamin_1994@126.com (Y.L.); yuanmiao@semi.ac.cn (M.Y.); wangyj@semi.ac.cn (Y.W.); hdchen@semi.ac.cn (H.C.); 2 University of Chinese Academy of Sciences, Beijing 100049, China; 3 Department of Ophthalmology, Peking University People’s Hospital, Beijing 100044, China; huijuanwu@vip.sina.com

**Keywords:** IOP, glaucoma, wearable, strain gauge, MEMS, high sensitivity

## Abstract

Intraocular pressure (IOP) is an essential indicator of the diagnosis and treatment of glaucoma. IOP has an apparent physiological rhythm, and it often reaches its peak value at night. To avoid missing the peak value at night and sample the entire rhythm cycle, the continuous monitoring of IOP is urgently needed. A wearable contact lens IOP sensor based on a platinum (Pt) strain gauge is fabricated by the micro-electro-mechanical (MEMS) process. The structure and parameters of the strain gauge are optimized to improve the sensitivity and temperature stability. Tests on an eyeball model indicate that the IOP sensor has a high sensitivity of 289.5 μV/mmHg and excellent dynamic cycling performance at different speeds of IOP variation. The temperature drift coefficient of the sensor is 33.4 μV/°C. The non-invasive IOP sensor proposed in this report exhibits high sensitivity and satisfactory stability, promising a potential in continuous IOP monitoring.

## 1. Introduction

Glaucoma is the second most prolific cause of irreversible blindness diseases in the world, and it is estimated that the number of people affected by glaucoma worldwide will reach 111.8 million in 2040 [1]. While elevated intraocular pressure (IOP) is no longer part of the definition of glaucoma, it is recognized as the only modifiable risk factor for the development and progression of the disease [2]. The accurate value of IOP is an important reference for the diagnosis and treatment of glaucoma. The IOP is a dynamically changing parameter, and the peak of IOP commonly occurs at night [3], which makes the diagnosis of glaucoma difficult. Continuous 24-h IOP monitoring is helpful for the early detection and timely intervention of patients with glaucoma [4]. For patients with early glaucoma, the use of IOP-lowering drops can control the IOP within the normal range for a period of time to slow down the progression of the glaucoma disease, but it is difficult to figure out the specific time of the medical treatment for different patients and find the best time for them, which makes it impossible for doctors to develop personalized treatment plans for patients and prevent the loss of the visual field of patients [5]. Therefore, the continuous real-time monitoring of IOP is of great significance for diagnosing and treating glaucoma.

The Goldmann applanation tonometer (GAT) is considered the “gold standard” for measuring IOP [6], and it is also the most commonly used method of measuring IOP in hospitals. However, the operation of GAT to measure IOP is complicated, and only intermittent IOP values can be obtained. It is impossible to fully understand the patient’s IOP fluctuations throughout the day with GAT, which makes the early diagnosis of glaucoma difficult. Therefore, there is an urgent need to develop portable, easy-to-operate, and low-cost devices that can continuously monitor IOP in real-time. The development of micro-electro-mechanical (MEMS) technology has promoted the widespread use of wearable medical electronic devices to monitor and treat human health physiological indicators [7,8], such as flexible electronic skin [9] and smart contact lens [10,11]. Researchers have proposed many IOP detection devices based on different principles to achieve continuous IOP measurement in recent years. According to the way the sensor is worn, IOP sensors can be divided into invasive and non-invasive types. Invasive IOP sensors [12,13,14,15,16,17,18] require the surgical implantation of pressure-sensitive elements inside the eyeball to measure IOP changes directly. The irreversible damage to the eyeball caused by implant surgery is still hard to accept for most people, which limits its clinical application. It is found that there is a certain correlation between corneal curvature and IOP. An IOP change of 1 mmHg will cause a change of central corneal curvature of about 3 um (for a corneal curvature of 7.8 mm) [19]. According to this feature, when the contact lens follows the cornea to deform conformally, the sensor integrated on the contact lens can detect the contact lens’ deformation to monitor IOP changes in real-time. Based on the sensing mechanisms, non-invasive IOP sensors are divided into two types, optical type [20,21,22,23] and electrical type [24,25,26,27,28,29,30,31,32,33]. Campigotto et al. built a microfluidic channel in a contact lens and injected dye reagents into the channel to sense the IOP [22]. When the IOP changes, the microfluidic channel volume will change with the deformation of the cornea, causing the dyeing reagents in the channel to move. The IOP changes can be monitored by reading the displacement of the dyeing reagent. Maeng et al. fabricated a photonic crystal film on a contact lens [23]. The photonic crystal film’s lattice structure changes with the variation in IOP, resulting in changes in the photonic crystal film’s peak reflection wavelength. The changes in the reflection wavelength peak were detected by a spectrometer. The optical IOP sensors integrated into the contact lens are relatively simple, but most of them require complex external equipment to amplify and read the optical signal. Additionally, it is difficult to achieve IOP detection when the eyelids are closed. Compared with optical methods, electrical methods of detecting IOP are less disturbed by eyelid movement. Electrical IOP sensors can be divided into three types by the sensing elements: capacitive type [24,25,26], inductive type [27,28], and piezo-resistive type [29,30,31,32,33]. In 2013, Chen et al. integrated a capacitive strain sensor into the contact lens [24], which contains an LC oscillator circuit composed of a curvature-sensitive capacitor and a toroidal inductor in series. The frequency response sensitivity measured by a vector network analyzer is 23 kHz/mmHg. In the same year, they fabricated an inductive strain IOP sensor [27], which achieved a sensitivity of 8 kHz/mmHg. They integrated a curvature-sensitive inductance coil and a capacitor in series to form an LC oscillation circuit. Either capacitive or inductive sensors require a frequency or phase discriminator to read the signals. The discriminator, similar to optical reading equipment, is not portable due to its large size, resulting in difficulty in achieving continuous monitoring of IOP in the clinic. Different from the optical or the LC oscillator sensor, the resistance strain sensor and its reading system can be miniaturized [30]. However, the sensitivity of the resistance strain IOP sensor is only 113 μV/mmHg. Increasing the sensitivity of the sensor will improve the precision and capacity of resisting disturbance. Besides this, it can reduce the demand for the reading precision of wireless reading devices to lower the cost.

In this work, our aim was to develop a non-invasive wearable contact lens sensor with high sensitivity, high linearity, and satisfactory stability for continuous IOP monitoring. The structure and parameters of the sensor were optimized by simulating the corneal deformation with COMSOL. The contact lens sensor was fabricated with a Pt strain gauge wrapped in a polyimide (PI) insulating layer, prepared by an MEMS process and packaged into the transparent polydimethylsiloxane (PDMS). The sensor showed a sensitivity of 289.5 μV/mmHg and high linearity of 0.9987, in the pressure range of 15–35 mmHg, in a simulated eyeball model test. Besides this, it exhibited an excellent dynamic cycling performance at different speeds of IOP changes. Therefore, the contact lens sensor proposed in this report is of high potential to be applied in continuous IOP monitoring under various physiological conditions.

## 2. Materials and Methods

**Figure 1:**
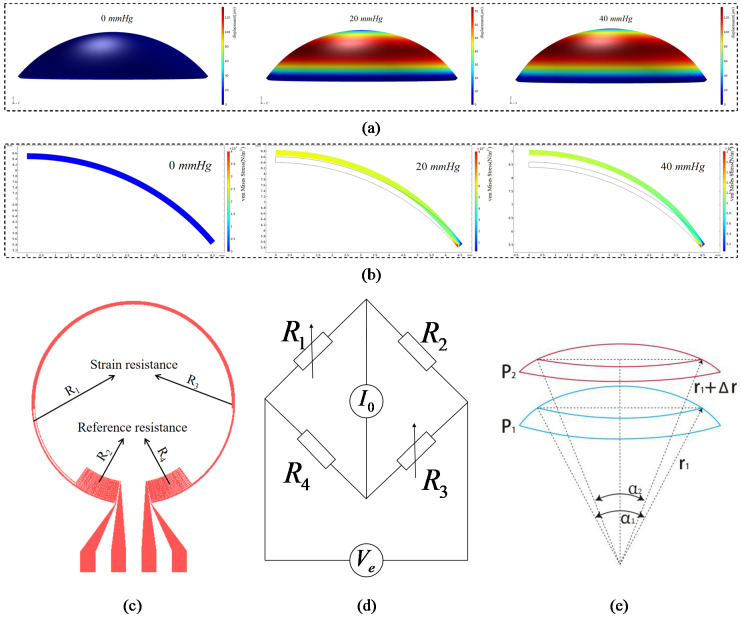
(**a**) Finite element simulation analysis of corneal deformation profile under the IOP of 0 mmHg, 20 mmHg and 40 mmHg. (**b**) Finite element simulation analysis of corneal von Mises stress distribution and deformation profile of cornea under the IOPs of 0 mmHg, 20 mmHg and 40 mmHg. (**c**) Strain gauge structure design diagram. (**d**) Wheatstone bridge schematic diagram. (**e**) Schematic diagram of contact lens deformation caused by changes in IOP, resulting in changes in the radius of curvature Δr.

To optimize the location and the structure of the strain gauge, the deformation of the cornea was analyzed by finite element analysis. A cornea model was constructed with COMSOL. The uniform pressures of 0 mmHg, 20 mmHg and 40 mmHg were applied to the inner surface of the eyeball, respectively, and the simulation results are shown in Figure 1a,b. The simulation results show that the corneal deformation is anisotropic and mainly concentrates in the circumference of 9–13 mm diameter (the corneoscleral junction position) of the cornea. Therefore, the strain gauge should be placed along the circumferential direction of the contact lens to maximize the strain sensitivity.

The strain gauge adopted the Wheatstone bridge structure to improve the sensitivity. The strain gauge was composed of two strain resistances (*R*_1_ and *R*_3_) and two reference resistances (*R*_2_ and *R*_4_), as shown in Figure 1c. The strain resistances were designed in a circular ring shape, located in the corneoscleral junction position, to deform the most. The reference resistances were designed in the radial direction, and remained almost unchanged when the IOP changed.

The four initial resistances could be designed to be the same value (*R*_1_ = *R*_2_ = *R*_3_ = *R*_4_) by using the same electrode length for each. To obtain the linear relationship between the output voltage and the resistance changes, a constant current source *I*_0_ was supplied to the Wheatstone bridge, as shown in Figure 1d. The output voltage caused by the strain gauge changes can be expressed as the following formula:

(1) $$V_{e}=\frac{\Delta R}{2}I_{0}\approx \rho \frac{c\pi \Delta d}{WH}I_{0}$$

where ΔR is the variation in strain resistance of the strain gauge, *ρ* is the resistivity of the strained material, *c* is the number of turns of the strain resistance, Δd is the changes in the radius of the strain resistance coil, and *W* and *H* are the width and thickness of the strain resistance, which are approximately considered as constant values under weak deformation. Changes in IOP can cause the contact lens’ deformation, but the arc length of the contact lens remains unchanged during the deformation process because there is no force tangentially to the lens, as shown in Figure 1e. Therefore, the changes of the strain gauge coil radius Δd can be expressed as the following formula:

(2) $$\Delta d=(r+\Delta r)\sin\left[\frac{\alpha r}{2\left(r+\Delta r\right)}\right]-r\sin\frac{\alpha }{2}$$

where *r* is the initial curvature radius of the contact lens, Δr is the changes in the cornea’s curvature radius, and *α* is the initial opening angle of the strain resistance. It can be seen that the relationship between Δd and Δr is not linear, but considering that Δr << *r*, Formula (2) can be simplified to:

(3) $$\Delta d\approx \left(\sin\frac{\alpha }{2}-\frac{\alpha }{2}\cos\frac{\alpha }{2}\right)\Delta r=m\Delta r$$

where *m* is a constant, equal to $\sin\frac{\alpha }{2}-\frac{\alpha }{2}\cos\frac{\alpha }{2}$. Variations in IOP can cause changes in the corneal curvature radius. The expression is as follows:

(4) Δr=nΔP

ΔP is the changes of IOP, and *n* is a constant, which depends on the eyeball mechanics’ biological characteristics, here *n* = 3 [19]. Derived from Formulas (1), (3) and (4), we can get:

(5) $$V_{e}\approx \frac{3\pi cm\rho I_{0}}{WH}\Delta P$$

The output voltage is approximately linearly related to the changes in IOP. Reducing the width *W* and thickness *H* of the strain gauge coil film, increasing *c* and *α*, and using metal materials with high resistivity *ρ* can improve the sensor’s sensitivity. The parameters of the designed sensor are shown in Table 1. The strain gauge was designed far from the center of the cornea to avoid the view being obstructed.

## 3. Fabrication

**Figure 2:**
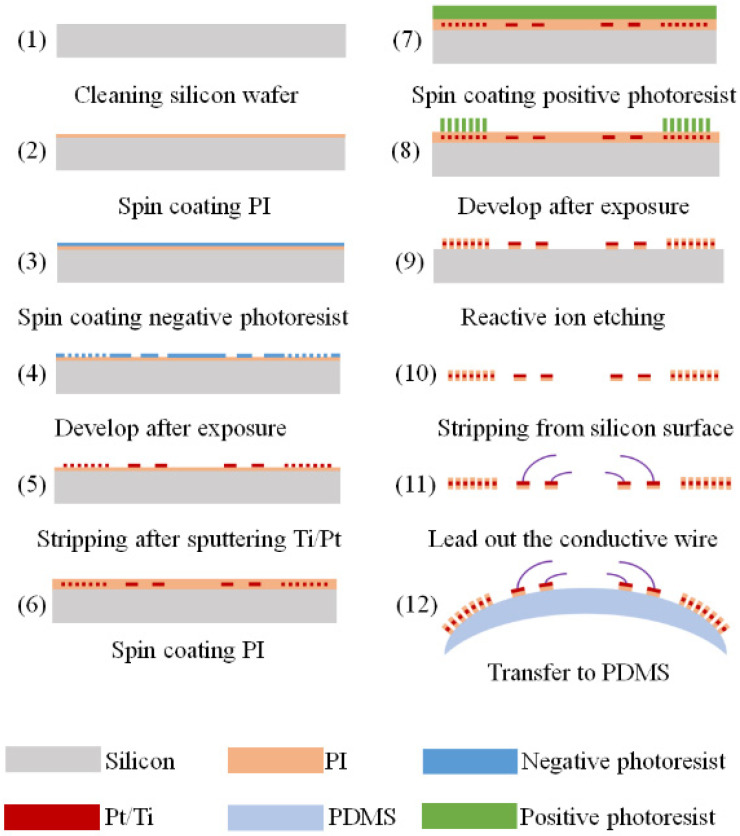
Process flow of sensor fabrication.

The sensor’s fabrication process is shown in Figure 2. (1) A four-inch silicon wafer was cleaned with acetone, absolute ethanol, and deionized water in sequence. (2) A 2 μm thick PI (bottom PI) was spin-coated on the silicon wafer and we employed curing by stepwise heating to 300 °C. (3) The negative photoresist (AR-N 4340) with a thickness of 1 μm was spin-coated onto the surface of the bottom PI and prebaked at 110 °C for 2 min. (4) A mask template was used to irradiate the photoresistor under an exposure machine. Then, the mask pattern could be formed on the surface of the bottom PI after baking and developing. (5) An oxygen plasma system was used to remove the residual photoresistor for 2 min, which is beneficial to the adhesion of a deposited metal layer to the PI substrate. Next, a titanium (Ti) layer with a thickness of 10 nm and a Pt layer with a thickness of 90 nm were deposited by thermal evaporation in sequence, and then the silicon wafer was stripped in an acetone solution to obtain the patterned metal film layer. (6) A 2 μm thick PI (top PI) was spin-coated on the surface of the metal layer. (7) A layer of 6 μm thick positive photoresistor (AZ 4620) was spin-coated on the top PI. (8) The positive photoresistor was exposed with a mask template under the exposure machine. After developing and post-baking, the designed patterns could be formed on the top PI. (9) The positive photoresistor was used as the masking layer. A reactive ion etching machine was used to etch the PI layer to acquire the strain gauge. (10) The strain gauge was peeled from the silicon wafer. (11) The electrode of the strain gauge was connected to the conductive wires by conductive silver glue. (12) PDMS (Sylgard 184, Dow Corning) with the weight ratio of prepolymer to curing agent of 10:1 was prepared. PDMS was cast onto the contact lens mold, and then the fabricated strain gauge was attached to the contact lens mold. After that, PDMS was dripped on the strain gauge again and cured at 90 °C for 2 h. Finally, a curved contact lens sensor was peeled off the mold.

**Figure 3:**
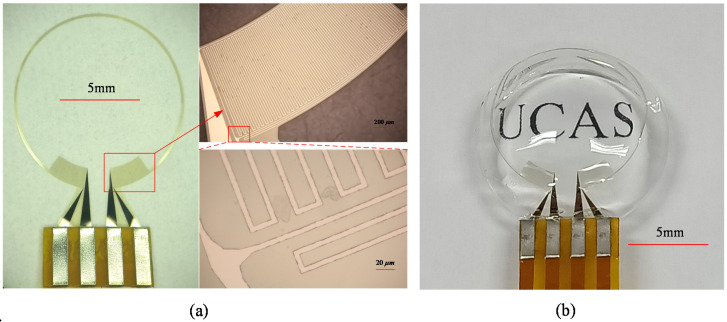
Physical image of (**a**) strain gauge and (**b**) contact lens sensor.

The strain gauge before being packaged into a PDMS-made contact lens is shown in Figure 3a. Because PDMS has the advantages of high permeability, transparency, flexibility and biocompatibility [34,35,36], the contact lens sensor has excellent flexibility and transparency, as shown in Figure 3b.

## 4. Testing

### 4.1. Setup of the Testing Systems

The impedance of the strain gauge was tested by a digital multimeter (Agilent U1241B). The Wheatstone bridge was powered by a source meter (Agilent B2902A), and the voltage changes caused by stress were detected and recorded by a source meter (KEITHLEY 2450) connected to a computer.

**Figure 4:**
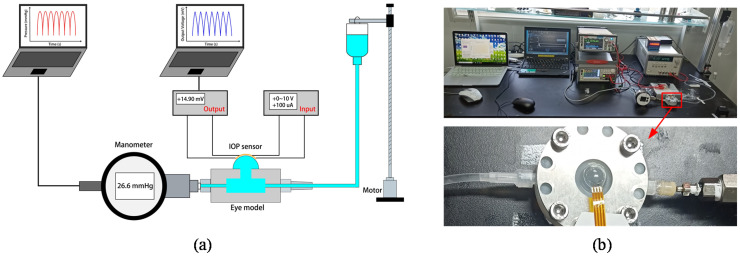
(**a**) Schematic diagram of the IOP test system. (**b**) Physical photo of the IOP test system.

Figure 4 shows the simulating eyeball test system. The silicone eyeball model was made with PDMS. The PDMS eyeball model was approximately a hollow sphere. The thickness of the sphere well was 200 μm, and the outer radius was 8.6 mm. The hollow sphere was filled with saline. An infusion bottle filled with saline and a liquid manometer was connected to the simulating eyeball. Changing the height of the infusion bottle could change the pressure of the simulating eyeball. A stepping motor was used to control the height of the infusion bottle. The liquid manometer was connected to a computer to record data. The contact lens sensor was attached to the simulating eyeball. A 100 μA constant current was supplied to power the sensor. The output voltage of the sensor was recorded with variations in the pressure.

### 4.2. Temperature Characteristics

To investigate the effect of temperature on the sensor, we tested the output voltage of the sensor working at different temperatures. The contact lens sensor was fixed on a glass plate and placed in an oven. The temperature was gradually increased from 32 °C to 36 °C.

When the sensor is working, the current will generate heat in the resistors. To verify the sensor’s safety on the human body, the temperature of the contact lens sensor was tested. Under an ambient temperature (24 °C), the heat generations were monitored by an infrared thermal imager (Fotric 226) during the running of the sensor with and without constant current source input.

## 5. Results and Discussion

**Figure 5:**
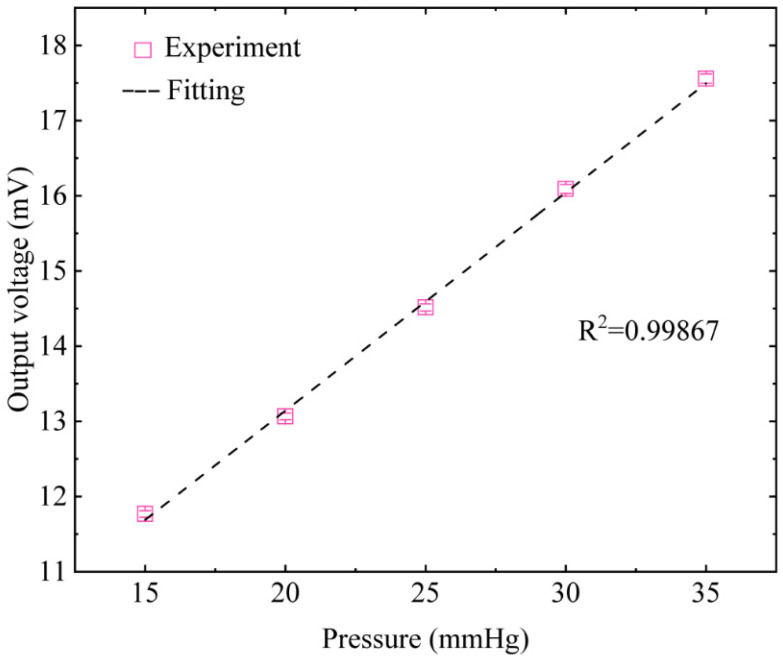
The response of the sensor’s output voltage to variations in silicone eyeball pressure.

The average resistance of each arm of the strain gauge is 27.6 ± 0.3 kΩ, and such a high impedance value can reduce the power consumption of the sensor. Figure 5 shows the corresponding output voltage value of the IOP sensor under different pressure values. Within the range of 15–35 mmHg, the sensitivity of the IOP sensor is 289.5 μV/mmHg. The correlation regression coefficient is 0.99867. The sensitivity is two times higher than the previous works on the same type of IOP sensors [30]. Table 2 compares the sensitivity of the proposed IOP sensor with other IOP sensors.

**Figure 6:**
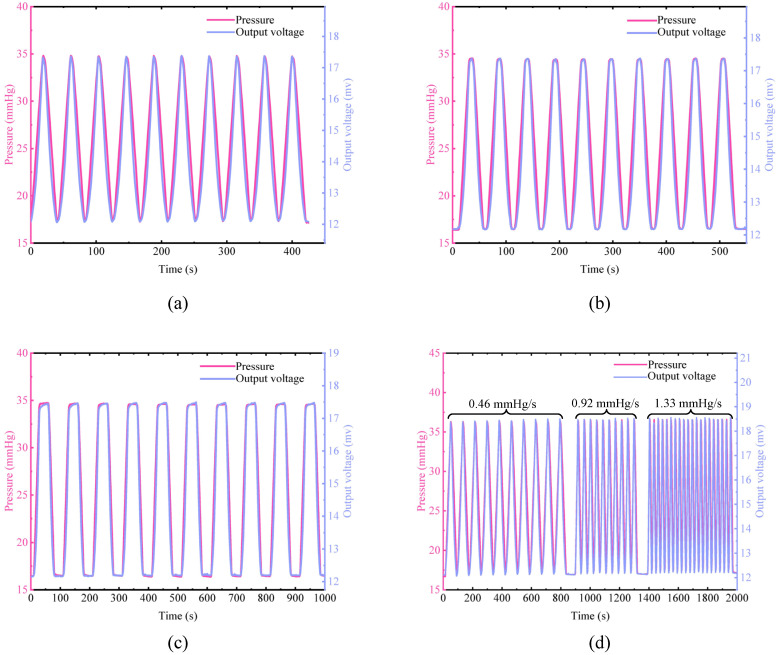
Dynamic performance of a contact lens sensor on the eyeball model. (**a**–**c**) The dynamic cyclic performance of the sensor under different pressures when staying for 0 s, 6 s, and 30 s at the peak, respectively. (**d**) The dynamic cycle performance of the sensor at different speeds of IOP variation.

**Figure 7:**
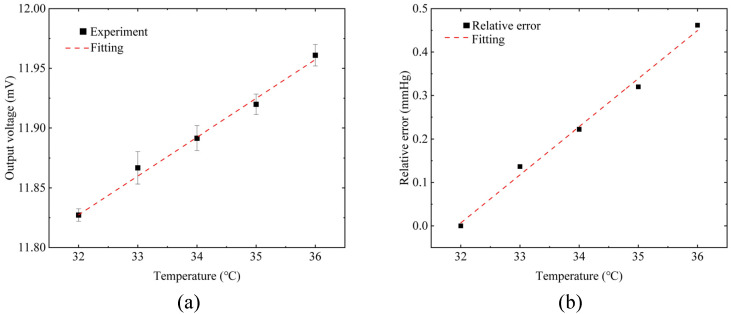
(**a**) Changes in the sensor’s output voltage when the temperature increases from 32 °C to 36 °C. (**b**) Related measurement error when the temperature increases from 32 °C to 36 °C.

The pressure of the simulating eyeball was controlled to perform multiple cycles of testing within the range of 16.5–35 mmHg. The output voltage versus pressure of the sensor is shown in Figure 6a. In the range of 16.5–35 mmHg, the average sensitivity of the sensor is 287.6 μV/mmHg. The pressure was maintained at the peak value for some time to further test the stability and response performance of the sensor. The results of the peak value lasting for 6 s and 30 s are shown in Figure 6b,c. When the pressure is maintained at the peak, the sensor output voltage is also maintained at the peak for the same time. The amplitude of the output voltage in the cyclic test has been kept constant and has satisfactory repeatability. These indicate that the sensor has high stability and can accurately detect static IOP. The sensor’s output voltage curve almost coincides with the pressure curve, indicating that the sensor has high linear responsiveness and can monitor pressure changes in real-time. The voltage output characteristics at different speeds of pressure variation were also tested, as shown in Figure 7d. When the pressure cycle test is performed with pressure change rates of 0.46 mmHg/s, 0.92 mmHg/s, and 1.33 mmHg/s, the voltage output amplitude hardly changes. The response speed can satisfy the pressure change ratio at 1.33 mmHg/s. This rate of IOP change rarely happens in humans. It proves that the sensor has excellent dynamic response characteristics and can monitor IOP changes at different speeds.

The zero-strain output voltage of the sensor at different temperatures is shown in Figure 7a. The sensor’s output voltage rises with the increase in temperature. It is worth noting that the output voltage of the sensor under zero-strain is about 11.83 mV. The strain gauge was deformed when it was packaged into the curved PDMS contact lens. The deformation causes the output voltage of the sensor at “zero-strain” to be 11.83 mV.

The rise in temperature will bring about changes in both the impedance and the strain of the Pt resistance. Although the temperature coefficient of the Pt impedance is as high as 3850 ppm/°C [37], the construction of the Wheatstone bridge can well eliminate the impact of temperature fluctuations. As far as the strain caused by the temperature is concerned, the bridge’s geometric structure is not symmetrically distributed on the contact lens. Compared with the reference resistances located near the center, the strain resistances are fabricated closer to the edge. We suppose that the rise in temperature causes different thermal strains on the PDMS-made contact lens, which changes the strain gauge, resulting in the voltage shift. The sensor’s temperature drift coefficient is about 33.4 μV/°C, and a temperature change of 1 °C can cause a measurement error of about 0.12 mmHg pressure, as shown in Figure 7b. The usual range of human corneal surface temperature is 32.9–36 °C [38], and the corresponding IOP fluctuation is 0.339 mmHg. The maximum IOP value caused by temperature fluctuation is no more than 0.4 mmHg, which satisfies the general accuracy requirements of the tonometer [39].

**Figure 8:**
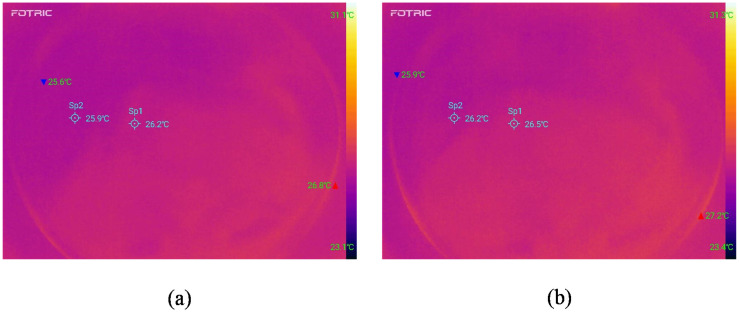
(**a**) Infrared thermal image of the sensor without current source. (**b**) Infrared thermal image of the sensor after working for 30 minutes under a 100 μA constant current source.

The heat generations of the sensor without current source input and working at a constant current source of 100 μA are shown in Figure 8. When the ambient temperature is about 24 °C, the maximum temperature without current source input is 26.8 °C in the sensor area. In comparison, the maximum temperature under the input of 100 μA constant current source for 30 min is 27.2 °C, so there is no noticeable temperature difference between them. It is proven that the contact lens sensor has low power consumption during working, and will not subject the human body to the harm caused by heat generation.

To achieve the wireless reading of IOP data, it is also necessary to design the application-specific integrated circuit (ASIC) combined with the radio frequency identification (RFID) technology. In addition, the sliding of the sensor at different positions of the cornea will produce a slight deviation in the sensitivity of the sensor, so the measurement data needs to be corrected by some related algorithms [40]. We will focus on the research of these problems in our next-step work.

## 6. Conclusions

In summary, this report presents the development of a non-invasive wearable contact lens sensor to monitor IOP changes in real-time continuously. A Wheatstone bridge composed of two strain resistances and two reference resistances was designed to improve the sensitivity and precision of the sensor. The design parameters of the sensor were optimized to further improve the sensitivity under the guidance of the simulation results of corneal deformation. The Pt strain gauge was fabricated by a MEMS process and packaged into the PDMS layer to form the contact lens sensor. The average sensitivity of the contact lens sensor is 289.5 μV/mmHg in the IOP range of 15–35 mmHg, which is higher than the previous works on the same type of IOP sensors. Moreover, it exhibits excellent repeatability and a dynamic response to different speeds of IOP changes. The temperature drift coefficient of the sensor is 33.4 μV/°C, which ensures that the measurement error of the sensor caused by temperature will not exceed 0.4 mmHg. Finally, the maximum temperature change of the sensor is only 0.4 °C when tested under a 100 μA constant current source for 30 minutes, which proves the sensor’s safety during wearing. As such, the IOP sensor proposed in this paper has broad application prospects in continuous IOP monitoring for glaucoma patients.

## Figures and Tables

**Table 1:** Sensor design parameters.

Number of strain resistance turns	3
Outer diameter of strain resistance	10 mm
Inner diameter of strain resistance	9.71 mm
Outer diameter of reference resistance	9.68 mm
Inner diameter of reference resistance	7.87 mm
Width of strain gauge	5 μm
Thickness of strain gauge	100 nm
The diameter of the contact lens	14 mm

**Table 2:** Performance comparison with previous reports.

Reference	Invasive or Non-Invasive	Method	Sensitivity
[12]	invasive	capacitance	160 kHz/mmHg
[16]	invasive	capacitance	15 kHz/mmHg
[22]	non-invasive	microfluidic	28 μm/mmHg
[24]	non-invasive	capacitance	23 kHz/mmHg
[27]	non-invasive	inductance	8 kHz/mmHg
[28]	non-invasive	inductance	35.1 kHz/mmHg
[29]	non-invasive	resistance strain	8.37 μV/mmHg
[30]	non-invasive	resistance strain	113 μV/mmHg
[32]	non-invasive	resistance strain	20 μV/mmHg
**This work**	**non-invasive**	**resistance strain**	**289.5 μV/mmHg**

## Data Availability

The data presented in this study are available on request from the corresponding author.
